# A pilot metagenomic study reveals that community derived mobile phones are reservoirs of viable pathogenic microbes

**DOI:** 10.1038/s41598-021-93622-w

**Published:** 2021-07-08

**Authors:** Matthew Olsen, Rania Nassar, Abiola Senok, Abdulla Albastaki, John Leggett, Anna Lohning, Mariana Campos, Peter Jones, Simon McKirdy, Lotti Tajouri, Rashed Alghafri

**Affiliations:** 1 Faculty of Health Sciences and Medicine, Bond University, Robina, QLD 4229 Australia; 2 Dubai Police Scientists Council, Dubai Police, Dubai, United Arab Emirates; 3 College of Medicine, Mohamed Bin Rashed University of Medicine and Health Sciences, Dubai, United Arab Emirates; 4 Oral and Biomedical Sciences, School of Dentistry, College of Biomedical and Life Sciences, Cardiff University, Cardiff, UK; 5 Harry Butler Institute, Murdoch University, Murdoch, WA 6150 Australia; 6 CSIRO Land and Water, CSIRO Health and Biosecurity, Floreat, WA Australia; 7 Dubai Future Council On Community Security, Dubai, United Arab Emirates; 8 General Department of Forensic Sciences and Criminology, Dubai Police, Dubai, United Arab Emirates; 9 Genomics and Molecular Biology, Bond University, Gold Coast, QLD 4229 Australia

**Keywords:** Risk factors, Infectious diseases

## Abstract

There is increasing attention focussed on the risks associated with mobile phones possibly serving as ‘Trojan Horse’ fomites for microbial transmission in healthcare settings. However, little is reported on the presence of microbes on community derived mobile phones which in 2021, numbered in the billions in circulation with majority being used on a daily basis. Identify viable microbial organisms swabbed from smartphones on a university campus. Entire surfaces of 5 mobile phones were swabbed and examined for their microbial content using pre-agar-based growths followed by downstream DNA metagenomic next-generation sequencing analysis. All phones were contaminated with viable microbes. 173 bacteria, 8 fungi, 8 protists, 53 bacteriophages, 317 virulence factor genes and 41 distinct antibiotic resistant genes were identified. While this research represents a pilot study, the snapshot metagenomic analysis of samples collected from the surface of mobile phones has revealed the presence of a large population of viable microbes and an array of antimicrobial resistant factors. With billions of phones in circulation, these devices might be responsible for the rise of community acquired infections. These pilot results highlight the importance of public health authorities considering mobile phones as ‘Trojan Horse’ devices for microbial transmission and ensure appropriate decontamination campaigns are implemented.

## Introduction

Microbial contaminated platforms, known as fomites, are objects or materials responsible for microbial transmission^1^. A 2010 study explored the presence of bacteria and viruses on different fomites in elementary classrooms^2^^.^ The common communal items identified as fomites and acting as reservoirs for bacteria were water fountain toggles, pencil sharpeners, keyboards, and faucet handles.

While multiple fomites are reported in several other studies, smartphones and mobile phones are major common fomite platforms that may be responsible for microbial transmission. As frequent “highly touched” platforms, smartphones raise microbial transmission concerns. The United States Center for Disease Control and Prevention (CDC) has outlined that up to 80% of all infectious diseases are transmitted via hands^3^. International public health authorities and infection control bodies only provide limited warnings relating to mobile phones as fomites that may be potential sources of viral, bacterial, and fungal transmission. These fomites particularly have a strong point of difference when compared to other common fomites. Both the intrinsic features of mobile phones and user habits appear to be optimal conditions for microbes to thrive on surfaces of phones^4^.

Evidence is growing that mobile phones are contaminated with microorganisms as reported in a scoping review published in 2020, suggesting that mobiles phones as fomites are bypassing current gold standard handwashing practices and are possibly responsible for the transmission of microbial agents and thus acting as a ‘Trojan Horse’^4^. In the same year, an Australian research team has shown that SARS-CoV-2, responsible for COVID-19, could be retrieved from the surface of mobile phones after an extended period of up to 28 days^5^. Recent research has found that front-line healthcare professionals utilise mobile phones regularly at work, including bathrooms, and 57% confirmed they never clean their devices^6^. In a recent Chinese study, positive presence of the SARS-CoV-2 virus was found on mobile phones of asymptomatic COVID-19 patients, a positive finding that was in correlation with faecal based viral presence in their stool and raising further concerns relating to the use of mobile phone in bathrooms^7^. Additionally, many studies have reported the presence of viable drug resistant organisms on mobile phones used in health care settings^4^. The study by Meadow et al., showed that 22% of the bacterial taxa present on fingers of mobile phone owners were also present on their respective devices^8^. A study of operating rooms and intensive care units sampled the hands and mobile phones of staff and identified evidence of bacterial contamination in 94.5% of the samples^9^. Of significance was the identification of *Staphylococcus aureus* including methicillin resistant species in both hands and mobile phones of healthcare workers (HCWs) raising concerns relating to infection transmission^10^.

There is limited scientific literature reporting microbial populations present on mobile phones from members of the general community. The popularity of mobile phones and smartphones is an ever-growing trend and now in the hands of billions of consumers. Mobile device use is reported to occur a minimum of four hours each day, with touching and handling of phones occurring hundreds of times each day^11^. Current reported studies in the scientific literature have largely been conducted using methodological limitations leading to limited characterisation of the global microbial diversity and identification of microbes on mobile phones^4, 8^. While other studies utilised sequencing technologies such as the 16 s rRNA sequencing, leading to mostly the identification of bacterial populations^12^.

In this pilot study, we aimed to provide further evidence that mobile phones within the community, picked randomly and in small number, are active fomites. Swabs samples were collected and applied to agar media for microbial growth. Shotgun metagenomics analysis was undertaken to identify all agar derived colonies.

## Methods

A pilot study sample size of five mobile phones were randomly selected from faculty and students at Bond University, Queensland, Australia, and swabbed using culture swab EZ II swabs (Becton Dickson) pre-moistened with sterile saline. For sample collection, gloves were worn and changed regularly to prevent any cross-contamination. All swabs and collection devices used were purchased sterile pre-packaged devices. The mobile phones were sampled front and back with swabs then kept in portable containers and transported immediately to the laboratory for processing. Additionally, the participants were invited to fill in a questionnaire regarding their lifestyle and habits associated with the use of their mobile phones.

### Swab plating and broth suspension

Each of the five (5) phones was swabbed and cultured on three (3) different types of media namely horse blood agar (HBA), MacConkey agar (MAC) and nutrient agar (NUT) for 48 h at 37 °C incubation condition. Post incubation, the five HBA derived colonies were collected, pooled together, and placed into broth liquid medium. Other pooling was performed for MAC and NUT respective derived colonies. Hence, three nutrient broth suspension tubes were generated with each detaining all specifically derived colonies from HBA, MAC and NUT agar plates, respectively. These three nutrient broth suspensions (HBA, MAC and NUT specific) were processed for DNA extraction and subsequent shotgun sequencing in NextSeq500 sequencers.

### DNA extraction

The preliminary step of the DNA extraction process involved the use of bead beating with 0.1 mm diameter glass beads (BioSpec Products, Bartlesville, OK USA) on a Powerlyser 24 homogenizer (Mo-Bio, Carlsbad, CA USA) at the Australian Centre for Ecogenomics (ACE), Brisbane, Australia. Briefly, samples were transferred to a bead tube and 800 µl of bead solution (Qiagen, Germantown, MD USA) was added and bead-beat for five minutes at 2000 rpm, then centrifuged at 10,000 g for one minute. Following the addition of 60 µl of cell lysis buffer, tubes were vortexed and then heated at 65 °C for 10 min (while mixing at 1000 rpm), then vortexed again for 30 s and stored at − 20 °C pending DNA extraction. Prior to DNA extraction, samples were thawed at room temperature; vortexed and centrifuged for one minute at 10,000 g. The resulting lysate was transferred to a new collection tube and DNA extraction carried out using DNeasy Powersoil Kit (Qiagen), as per manufacturer protocol with a final elution volume of 50 µl using sterile, EDTA-free elution buffer.

### Metagenomic sequencing and bioinformatic analysis

Libraries were prepared according to the manufacturer’s protocol using Nextera DNA Flex Library Preparation Kit (Illumina San Diego, CA USA). Preparation and bead clean-up were run on the Mantis Liquid Handler (Formulatrix) and Epmotion (Eppendorf) automated platform. On completion of the library prep protocol, each library was quantified, and quality control (QC) was performed using the Quant-iT™ dsDNA HS Assay Kit (Invitrogen, Carlsbad, CA USA) and Agilent D1000 HS tapes on the TapeStation 4200 (Agilent Technologies, Santa Clara, CA USA) as per manufacturer’s protocol. Library Pooling, QC and Loading Nextera DNA Flex libraries were pooled at equimolar amounts of 2 nM per library to create a sequencing pool. The library pool was quantified in triplicates using the Qubit™ dsDNA HS Assay Kit (Invitrogen). Sequencing was carried out on the NextSeq500 (Illumina) using NextSeq 500/550 High Output v2 2 × 150 bp paired end chemistry according to manufacturer’s protocol^13^. The post-sequencing derived raw data were retained and transferred into Illumina base space platform (https://basespace.illumina.com). Following the sequencing runs, data as demultiplexed FASTQ files were uploaded into CosmosID platform (https://www.cosmosid.com/). Raw datasets Fastq files were analysed using the CosmosID software to identify bacteria, protists, bacteriophages, viruses, fungi, virulence factor genes and antibiotic resistance genes.

The CosmosID bioinformatics software package utilises a high-performance data-mining K-mer based algorithm that disambiguates hundreds of millions of short reads of a metagenomic sample into the discrete microorganisms engendering the particular sequences. Similarly, the collection of VFGs and ARGs in the microbiome was also identified against curated VFGs and ARGs in the databases. The overall database is derived from curated GenBook® Databases comprising over 150,000 bacteria, viruses, fungi, and protists genomes and gene sequences from both private and public sources such as NCBI/RefSeq/WGS/SRA/nr, PATRIC, M5NR, IMG, ENA, DDBJ. Data were filtered using a multi-kingdom resolutive taxonomic identification analysis built into CosmosID. This filtering was based on internal statistical scores from CosmosID, which enabled listing of results without further validation to determine their presence in the sample.

### Relative abundance calculation of specific microbe

The relative abundance is the percentage of a specific identified microbe in a microbial data category divided by the total amount of microbes within this same data category (times 100).

### Ethics

Ethical approval was obtained from Bond University Human Research Ethics Committee (16,004). All methods were carried out in accordance with relevant guidelines and regulations. Additionally, informed consent was obtained from all participants and none of them were under 18 years of age.

### Funding support

Funding for the DNA sequencing was made available thanks to a consultation research-based account owned by LT and administered at Bond University.

## Results

### Culture-based identification

**Figure 1:**
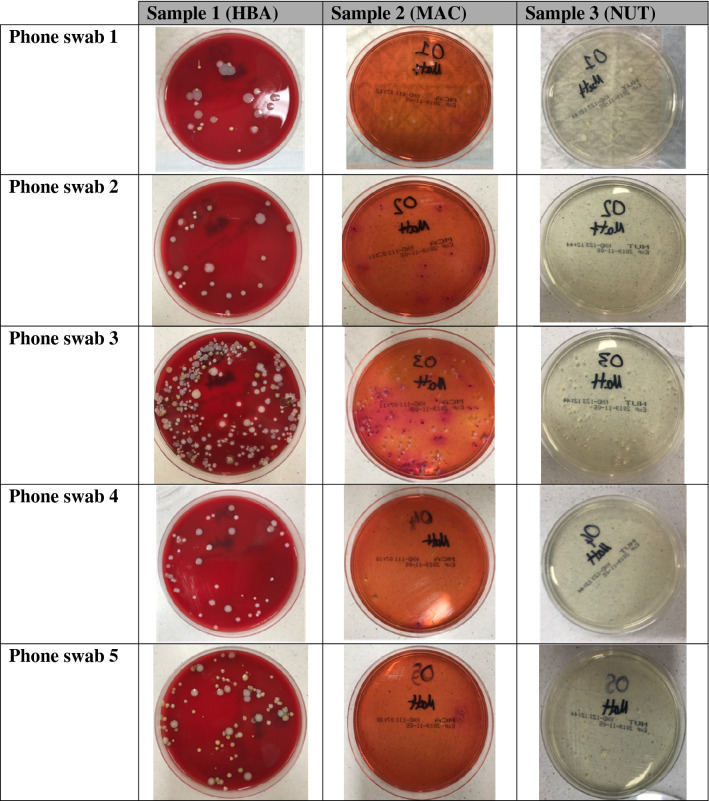
Sample growths of 5 community derived phone swabs on three different agar plates (HBA, MAC, NUT).

All phones were found to be contaminated with bacteria as shown by bacterial growth following 48 h of culture (Table 1). Phone swab 3 with the most significant growth across all three media plates (Fig. 1).

**Table 1:** Number of individual agar plate derived colonies retrieved from each phone swab.

	HBA	MAC	NUT	TOTAL
Phone 1	24	6	11	41
Phone 2	24	17	8	49
Phone 3	280	145	56	481
Phone 4	50	7	3	60
Phone 5	95	4	8	107

A total of 15 agar plates (5 HBA, 5 MAC and 5 NUT) were used to grow swabs derived from community phones (n = 5). Pooled colonies for HBA, MAC and NUT accounted for 473, 179 and 86 colonies respectively (Each pool was subject for downstream shogun sequencing).

### Sequencing output

The three pooled microbial entire colonies corresponding to the 5 HBA agars, the 5 NUT agars and the 5 MAC agars respectively were subject to three distinct next generation sequencing runs. The total sequencing reads of sample 1 (HBA specific), sample 2 (MAC specific) and sample 3 (NUT specific) was 41034,13842,162910, 53830216, respectively. All raw data were uploaded to the NCBI-SRA database under the accession number of Accession: PRJNA727685, ID: 727685. Global raw sequencing hits, not representing the diversity of each taxonomic entity, of microbial taxa showed bacteria representing the largest number of micro-organisms identified in this study followed by bacteriophages (53), fungi (8) and protists (8). Regarding hits associated with microbial genes, virulence factor genes and distinct antibiotic resistance genes accounted for 317 and 41, respectively.

### Microbial identification

#### Bacteria

**Figure 2:**
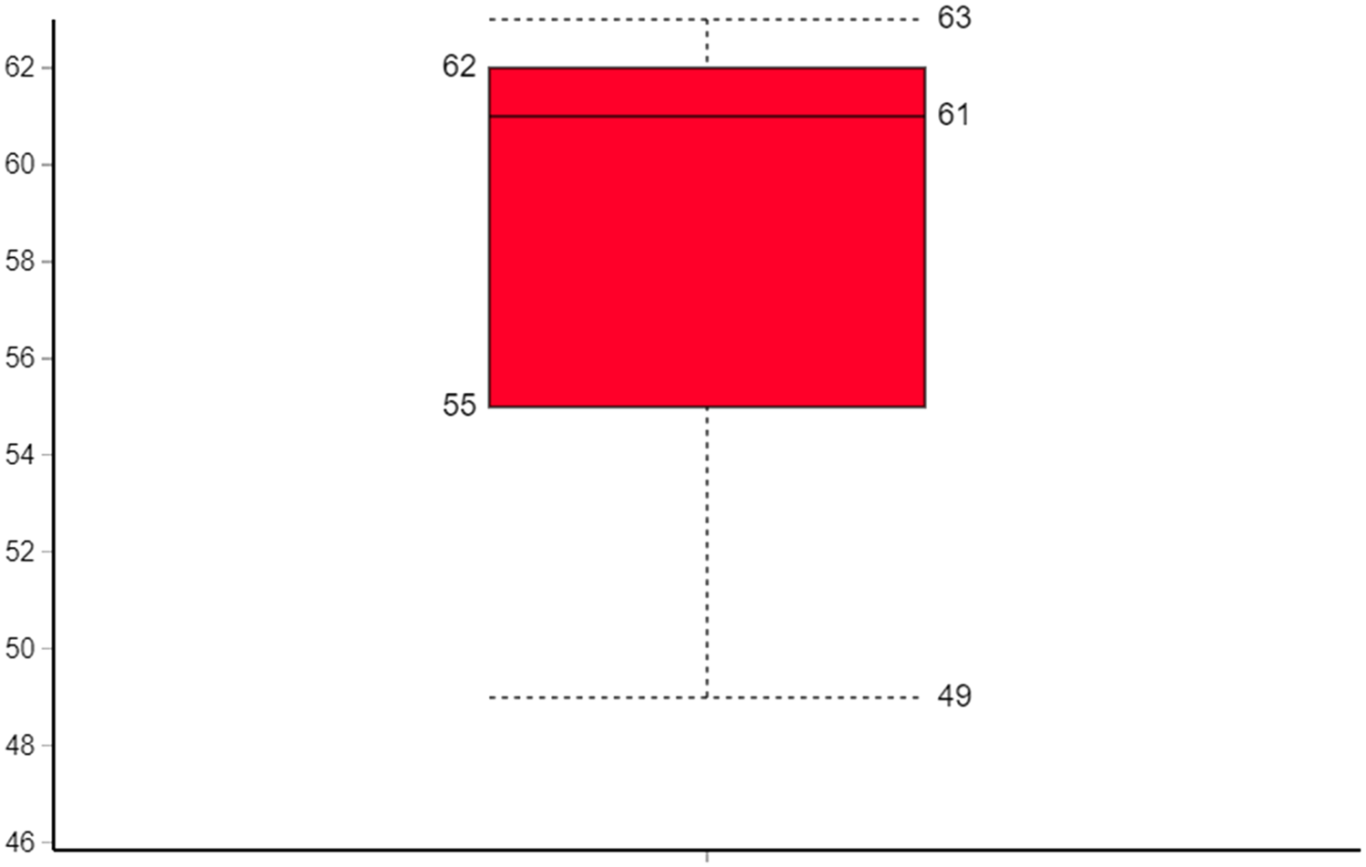
CHAO1 bacterial alpha-diversity representation across all three samples.

**Figure 3:**
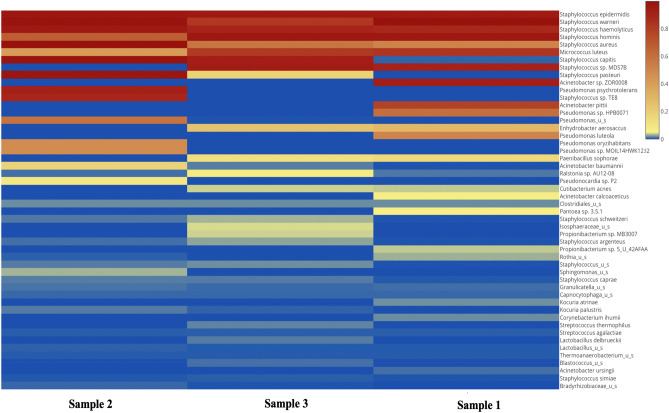
Heatmap visualisation of bacterial strains identified from community derived phone swabs.

173 different strains were identified of which 68.2% (118) were Gram-positive and 31.8% (55) Gram-negative bacteria. The list of all bacteria identified across the 5 community mobile phones is available in Appendix A (from metagenomes 1–3**)**. The alpha diversity of bacteria found across all three metagenomes is outlined in Fig. 2. Figure 3 illustrates a heatmap representation outlining the extended range of bacterial strains identified on community-derived mobile phones.

Of note, *Coagulase-negative Staphylococci* (CONs) represented 41.52% (49/118) of the total Gram-positive bacteria identified. Other noteworthy Gram-positive bacteria include *S. aureus, L. monocytogenes and B. cereus* accounting for 1.69% (2/118), 1.69% (2/118) and 0.84% (1/118) respectively. Additionally, *Streptococcus sp.* 4.23% (5/118) and *Enterococci spp.* 0.84% (1/118) were found on the swabbed phones.

Among the Gram-negative bacteria some pathogens were found and include *Acinetobacter baumannii* 5.45% (3/55) and *Pseudomonas aeruginosa* 3.63% *(*2/55). Of note, other *Pseudomonas* and *Acinetobacter* species accounted for 12.75% (7/55) and 12.72% (7/55) of the total Gram-negative bacteria, respectively. Additionally, faecal associated pathogenic Gram-negative bacteria were also identified and include *Salmonella enterica* 3.63% (2/55), *Bordetella pertussis* 1.81% (1/55), *Campylobacter* 1.81% (1/55) and *Escherichia coli* 1.81% (1/55).

#### Fungi and protists

**Figure 4:**
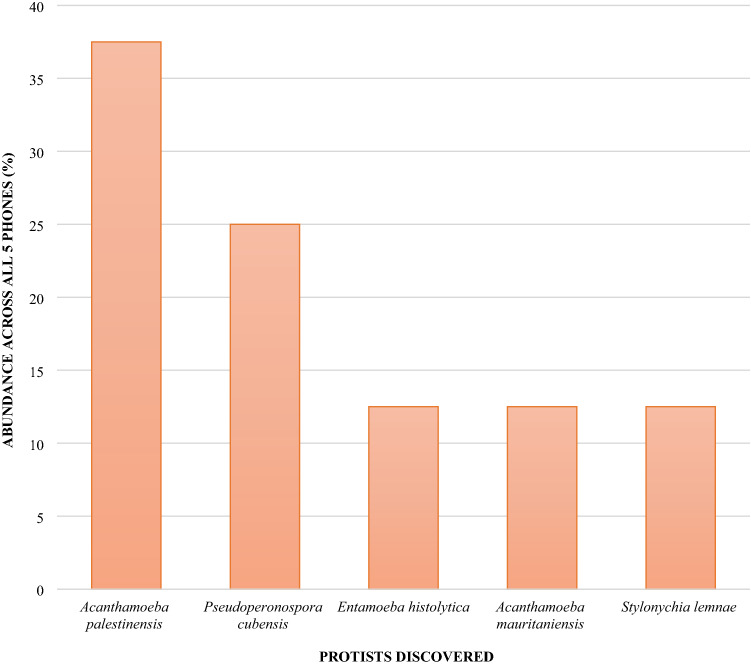
Relative abundance of protists identified on community derived phones.

Eight different fungal species were identified with the most prevalent species found being *Malassezia restricta* (25%) but with no human pathogenic fungi discovered in this study. Additionally, 5 different protists were found on mobile phones with human pathogens belonging to the protozoal group Sarcodina (Fig. 4).

#### Bacteriophages

**Figure 5:**
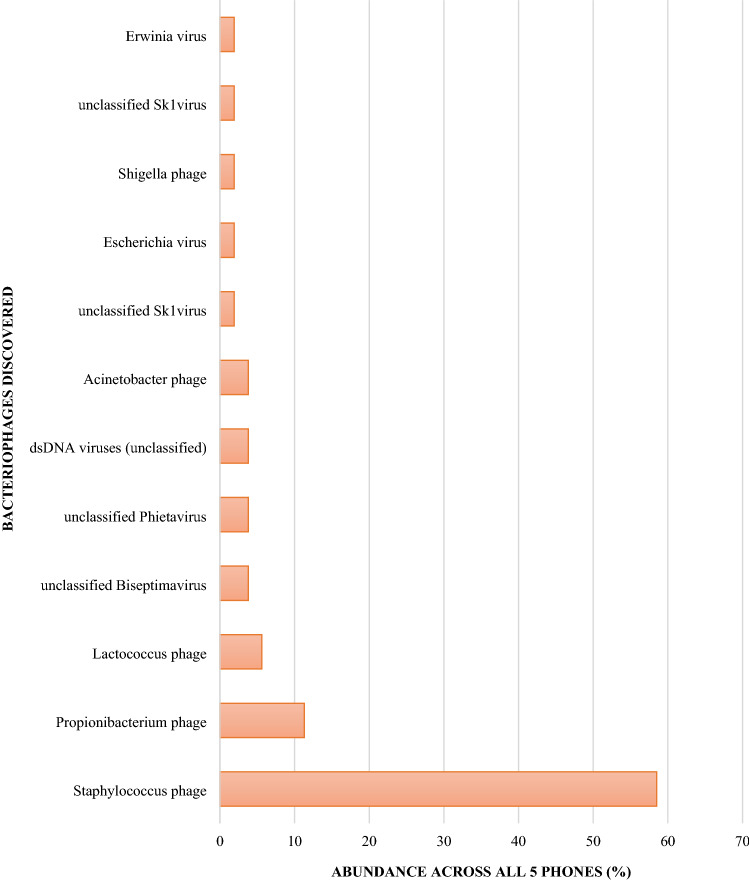
Relative abundance of bacteriophages, relating to specific bacteria, identified on community derived phones.

Most of the bacteriophages identified were related to *Staphylococcus* species (58.50%), followed by *Propionibacterium* phage (11.30%) and *Lactococcus* phage (5.60%) (Fig. 5).

### Virulome and resistome

#### Virulence factor genes

**Figure 6:**
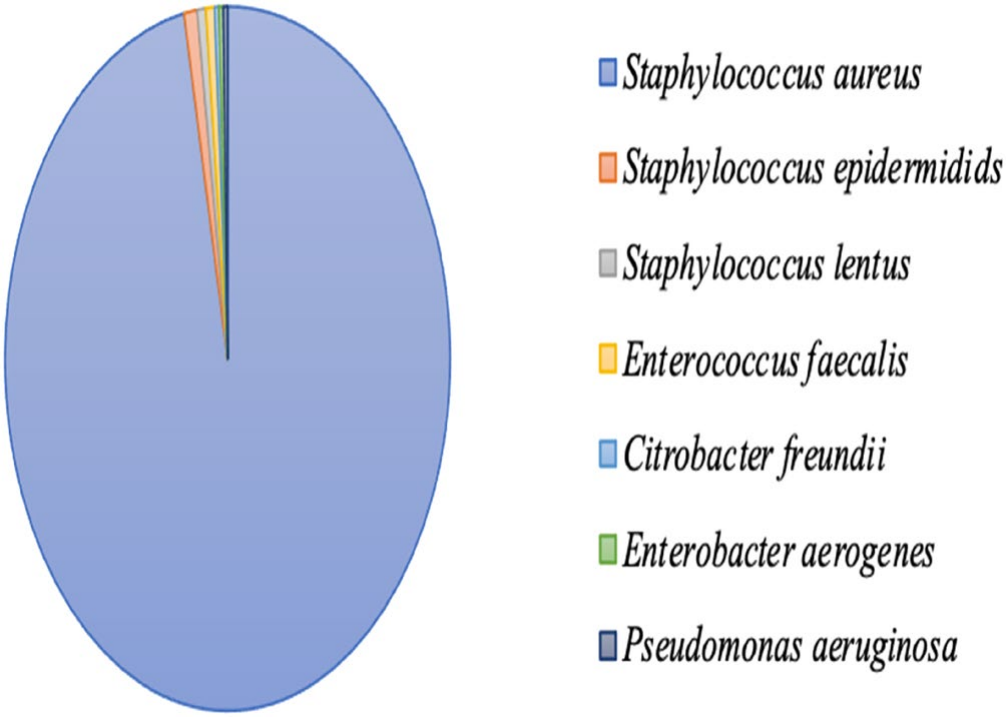
Relative abundance of virulence factor genes identified on community derived phones.

317 virulence factor genes were identified. The majority of these found to be associated with *S. aureus* (96%) (Fig. 6).

#### Antibiotic resistance genes

**Figure 7:**
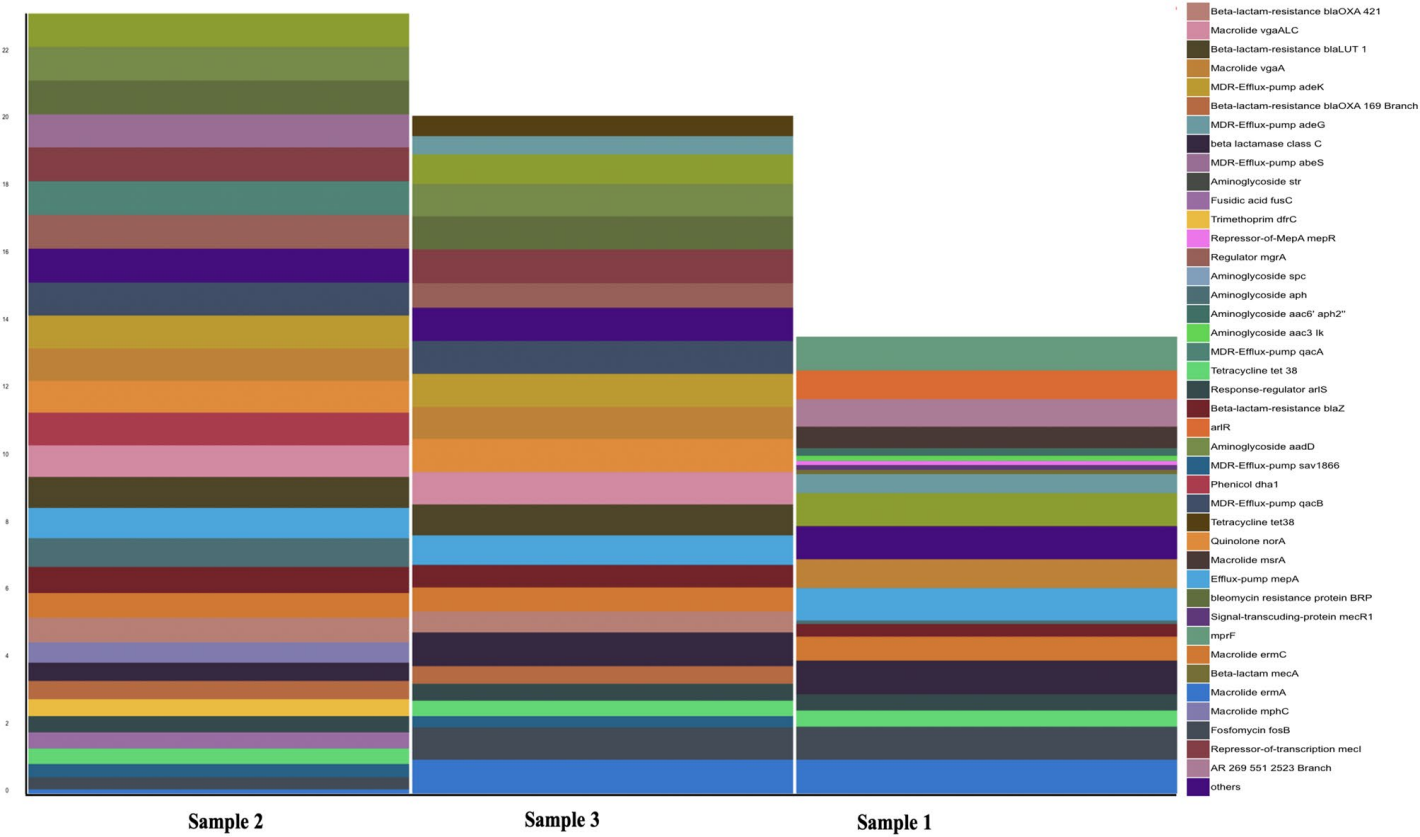
Individual metagenome distribution of identified antibiotic resistance genes from community derived phone swabs.

A total of 41 distinct antibiotic resistant genes were identified across all three metagenomes. Sample 1, 2 and 3 contained individually a number of 22, 30 and 25 ARGs respectively. the most common ones being MDR-Efflux-Pump inhibitors (14.28%), Beta-Lactam (12.98%), Macrolide (12.98%) and Aminoglycoside (10.38%) (Fig. 7).

#### Habits and lifestyle of participants

**Figure 8:**
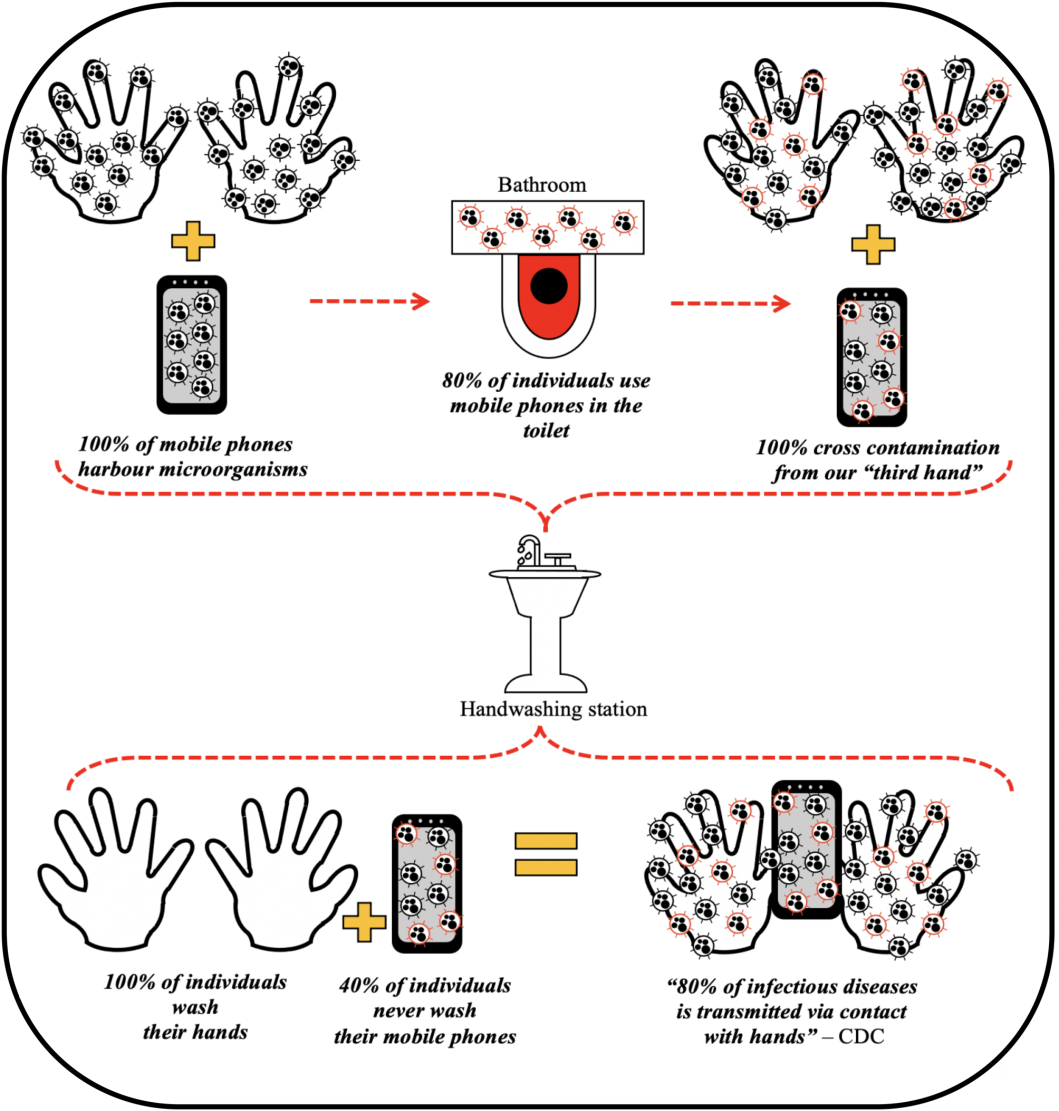
Infographic representation_Mobile phones serving as ‘Trojan Horse’ bypassing the current gold standard handwashing practices.

Participant's questionnaires identified that all 5 participants agreed that mobile phones harbour microbes but 40% confirmed they never wash their mobile phones and 40% confirmed they have washed their devices but not recently (within the past year). Of note, 4/5 mobile phone owners admitted using mobile phones in the bathroom. Two of these participants further admitted to never washing their devices (Fig. 8).

### Discussion

The combination of culture-based and global metagenomic sequencing undertaken in this pilot study has shown the presence of significant species and strain diversity on community derived mobile phones. The study demonstrated the presence of protists which have not been captured in previous reports. In addition, the metagenome of the microbial species isolated demonstrates an abundance of bacteriophages as well as antibiotic resistance and virulence genes. Despite this pilot study having a limited number of swabbed phones, we did successfully demonstrate that our method and subsequent metagenomic analysis of phone swabs are an effective means to identify a plethora of microbes on the surface of these devices.

This study found 173 different bacterial species across the five mobile phones subjected to the sequencing analysis. Most bacteria found are normal biota found on humans which include a large proportion of coagulase negative staphylococci with similar reported findings from other studies^14^.

Of concern is the identification of bacteria identified as ‘ESKAPE’ type. These included bacteria such as *S. aureus*, *A. baumannii*, *P. aeruginosa*, *Enterobacteriaceae sp.* and *Enterococci* organisms known for their pathogenicity and rising antimicrobial resistance. The presence of these bacteria on mobile phones in the community is concerning particularly for susceptible individuals. Of importance, such ‘ESKAPE’ bacteria are considered high priority concerns in hospital and other healthcare settings for their high contribution to nosocomial diseases. In a study^15^, this research team has been able to swab and demonstrate that these microbes are viable and present on the surface of mobile phones being used regularly by hospital staff in the workplace. The pathway for potential microbial transmission from healthcare settings to the community environment may be a reality; a hypothesis argumentatively plausible with non-existent phone decontamination disinfection protocols in place in most hospitals and staff bringing home contaminated phones with daily hospital derived microbes^4^

As stated above the findings of this pilot study identified the presence of *S. aureus*, a bacterium known for its high virulence, resistance to antibiotics and omni-presence member of ‘ESKAPE’ bacteria in hospitals. Strikingly, our study showed that 96% of virulence genes present on phones were associated with *S. aureus*. Furthermore, coagulase negative (CONs) staphylococci were the most numerous bacteria found on the studied phones. Interestingly, this high number of staphylococci bacteria was correlated with the high number of bacteriophages specifically targeting such bacteria on phones (58.5% of all bacteriophages). Another ‘ESKAPE’ derived bacterial finding was *A. baumannii*, a Gram-negative opportunistic micro-organism responsible for hospital associated infections (HAIs) affecting mostly prolonged hospital stay-patients (> 90 days) and immunocompromised individuals^12, 16^. Additional species of the Acinetobacter family were found in this study and included *A. nosocomialis, A. oleivorans, A. pittii* and *A. ursingii. P. aeruginosa* was also found on the swabbed phones. It is a bacterium commonly responsible for nosocomial diseases and recorded as containing high antibiotic resistance^17, 18^. Other species in the same genus were found and included *P. luteola* and *P. oryzihabitans,* both of which are involved in several disorders including endophthalmitis, peritonitis, sepsis, and bacteraemia with most susceptibility in the frail and infants^19, 20^.

Pathological faecal-based Gram-negative bacteria were identified. The presence of *Salmonella enterica* 3.63% (2/55), *Bordetella pertussis* 1.81% (1/55), *Campylobacter* spp 1.81% (1/55) and *Escherichia coli* 1.81% (1/55) is of concern.

While the sample size was small (5) the results of this study highlight that mobile phone’s hygiene is not high and represents a potential risk for disease transmission. Not only did a high proportion admit to using their phones in bathrooms but the number who admitted to never washing their phones was also high. With the high use rate of phones on a daily basis and the less than hygienic practices of users it is reasonable to expect that cross contamination of microorganisms from phones to hands will occur. Couple this with the CDC warnings that up to 80% of all infectious diseases are transmitted via hands and this study results further emphasize the risk posed by mobile phones (Trojan horses)^3, 4^ Not washing mobile phones is negating the value achieved through the current gold standard handwashing practices.

Billions of phones are used daily in the community for leisure, or during work including in the food industry (in the hands of food handlers) from fast food providers, restaurants to boat cruise buffets and global culinary professions such as traiteurs. Identifying the original causes of infection outbreaks within this sector is always a challenge. The handling of microorganism contaminated phones by workers, often with gloves on, should be carefully examined as it may be the potential etiological factor causing infections.

*B. cereus*, a Gram-positive spore-forming bacterium has been reported to cause a self-limiting food-poisoning syndrome characterised by diarrhoea/abdominal pain and/or nausea/vomiting (diarrheal type and emetic type) was isolated in this study. In addition, *B. cereus* can cause non-gastrointestinal diseases such as endocarditis, endophthalmitis and in rare cases lower respiratory-tract infections^21^. The natural reservoir of *B. cereus* includes decaying organic matter, water environments and fomites^22^, with research highlighting that *B. cereus* in food products are frequently ingested forming part of the transitory intestinal flora that are shed subsequently by carriers^23^. Emerging evidence is recognising the importance *B. cereus* as a pathogenic organism with the potential to lead to fatal outcomes^24^. Similarly, to *B. cereus*, *Clostridium* species was another spore-forming bacteria found on phones. These species are normally found in soil and can cause infection though skin abrasion, puncture wounds or ingestion of contaminated food products. By means of enterotoxins and neurotoxins production, *Clostridium spp.* can cause gastroenteritis and neuronal dysfunction. *Listeria monocytogenes* and *campylobacter spp* were two additional microbes found on the surface of the mobile phones within this study. These bacteria are associated with important gastro-intestinal infections^25^.

The finding of faecal microbes on mobile phones in this study is not unexpected as people have a habit to use their smartphones/mobile phones in restrooms. Contamination of mobile phones with faecal bacteria has been previously reported in other studies^4, 25^. Whilst individuals might or might not wash their hands when exiting restrooms, their phones used in toilets are likely to be contaminated either due to the flushing plume effect or simply by contact with yet un-washed hands. Coincidentally, a recent study working on the SARS-CoV-2 virus pandemic, showed that coronaviruses were retrieved from anal source samples belonging to asymptomatic COVID-19 positive patients and such viruses have been detected on mobile phones^7^. It had been previously hypothesised that mobile phones should be considered as a ‘Trojan Horse’ for SARS-CoV-2 virus and contributing to the transmission and spread of the disease globally^4^. Such phones would be contaminated because of viral shedding from COVID-19 sufferers either by faecal material deposition on phones, and/or contact of phones with uncleaned virally infected hands and/or patient’s deposition of high loads of droplet rich viruses during calls on mobile phones^7^. Indeed, a recent article demonstrated clearly that viruses (including SARS-CoV-2) can survive on glass surfaces (e.g., mobile phones) and polymer plastic surfaces (e.g., bank notes) for extended periods [up to 28 days in comparison to the previous estimated survival time of 14 days]^5^. While this current research did not aim to isolate viruses, its outcomes coupled with previous results, clearly demonstrates the need to undertake further research regarding mobile phones as a vehicle of SARS-CoV-2 transmission globally.

Furthermore, it is particularly interesting that this study has also found protozoa with a great representation of Sarcodina eukaryotic parasites such as *Acanthamoeba* species and *Entamoeba histolytica*. *E. histolytica* are associated with intestinal infections and extra-intestinal infections such as amoebic brain encephalitis^26^. These results highlight that with the high rate of touch for mobile phones linked with a user’s tendency to touch their faces up to 23 times an hour^27^ there is the opportunity for parasites to gain access to the user’s mouth, nose, or eyes.

*Bordetella pertussis* was also identified in our metagenomics study and this microbe is responsible for severe infections in children. Mobile phones may pose a risk for the transmission of whooping cough especially to areas with anti-vaccination communities or exposure of such contaminated phones with direct or indirect contact to susceptible babies under 6 months (not yet vaccinated against this bacterium). The overall results of this pilot study demonstrate the need for further investigations on the role mobile phones play as fomite surfaces and transmitters of pathogenic microbes in the community and globally.

Finally, our study found a large antimicrobial resistome profile with 41 distinct antibiotic resistant genes identified from the 5 phones swabbed. A 2015 study highlights the growing crisis that antibiotic resistance poses to healthcare systems worldwide^28^. In the same year, The United Nations General Assembly has identified 17 Sustainable Development Goals (SDGs)^29^ with the SDG number 3 particularly dedicated to health as “Good Health and Wellbeing”. The UN expects achieving all SDGs by 2030 however, SDG3 might be impossible since humanity is facing an unpreceded dual challenge: the rise of Antimicrobial resistance organisms (AMROs) and the discovery void of new efficient antibiotics.

## Conclusion and perspective

Mobile phones are microbial contaminated fomites and potential sources of microbial spread. Research focussed on this topic is limited but slowly gaining momentum because of its biosecurity relevance. The complex relation of mobile phones and humans: the in-built temperature control of phones; unhygienic hands frequently in contact with these devices; use of mobile phones in toilets and other less hygienic settings; the deposition of nutrients onto phone surfaces while eating; deposition of saliva droplets on phone surfaces during phone calls; and the paramount lack of any defined procedures to decontaminate these phones; has created a situation where it is not surprising that high touch screen devices, especially mobile phones, are ideal fomites for micro-organisms and are most probably contributing to global microbial dissemination.

Scientifically validated disinfection and decontamination strategies for mobile phones, and similar devices, must be implemented to achieve adequate disinfection of mobile phones. Despite current gold standard handwashing practices being well accepted by the community the frequent manual touching of uncleaned phones is bypassing the sanitation standard. There are billions of mobile phones in circulation worldwide and these are likely to be acting as potential ‘Trojan Horses’ for microbial spread across all sectors including the medical, hospitality and food industries.

To provide some current perspective, the COVID-19 pandemic’s rapid transmission of SARS-CoV-2 virus is still challenging the scientific community. While evidence of exposure to droplets, aerosols and physical direct or indirect contacts are confirmed pathways for transmission of the pathogen, little attention is being given to the role mobile phones are playing as fomites. These mobile devices are crossing international borders and continents totally unchecked for microbial contamination. This poses a yet unconfirmed, biosecurity pathway. With the recent emergence of SARS-CoV-2 new variants [United Kingdom (B.1.1.7 or Alpha variant), South African (B.1.351 or Beta variant), Brazilian P.1 (B.1.1.28.1 or Gamma variant) and the double mutant (E484Q and L452R) of the Indian variant B.1.617.2 or Delta variant] and their dissemination across the world in a short period of time, the hypothesis that mobile phones are silent but demonstrated fomite carriers of viable micro-organisms should trigger further research.

## Supplementary Information

Supplementary Information.
