# Supplementary material for: A pilot metagenomic study reveals that community derived mobile phones are reservoirs of viable pathogenic microbes
Source: Sci Rep. 2021 Jul 8;11:14102. doi: 10.1038/s41598-021-93622-w (PMC8266881; doi:10.1038/s41598-021-93622-w)
Supplement: Supplementary file 1 — Supplementary Information. [file 41598_2021_93622_MOESM1_ESM.docx]

**Sample 1 – Raw Data (Strain Level) – Total**

| **Name** | **Tax ID** | **Gram-Stain** |
| --- | --- | --- |
| Staphylococcus epidermidis | [1282](https://www.ncbi.nlm.nih.gov/Taxonomy/Browser/wwwtax.cgi?id=1282) | + |
| Staphylococcus warneri | [1292](https://www.ncbi.nlm.nih.gov/Taxonomy/Browser/wwwtax.cgi?id=1292) | + |
| Staphylococcus sp. MDS7B | [1209359](https://www.ncbi.nlm.nih.gov/Taxonomy/Browser/wwwtax.cgi?id=1209359) | + |
| Micrococcus luteus | [1270](https://www.ncbi.nlm.nih.gov/Taxonomy/Browser/wwwtax.cgi?id=1270) | + |
| Acinetobacter sp. ZOR0008 | [1339229](https://www.ncbi.nlm.nih.gov/Taxonomy/Browser/wwwtax.cgi?id=1339229) | - |
| Acinetobacter pittii | [48296](https://www.ncbi.nlm.nih.gov/Taxonomy/Browser/wwwtax.cgi?id=48296) | - |
| Staphylococcus haemolyticus DNF00585 | [1401071](https://www.ncbi.nlm.nih.gov/Taxonomy/Browser/wwwtax.cgi?id=1401071) | + |
| Pseudomonas sp. HPB0071 | [1203578](https://www.ncbi.nlm.nih.gov/Taxonomy/Browser/wwwtax.cgi?id=1203578) | - |
| Staphylococcus hominis subsp. hominis ZBW5 | [1185421](https://www.ncbi.nlm.nih.gov/Taxonomy/Browser/wwwtax.cgi?id=1185421) | + |
| Pseudomonas luteola XLDN4-9 | [1207076](https://www.ncbi.nlm.nih.gov/Taxonomy/Browser/wwwtax.cgi?id=1207076) | - |
| Enhydrobacter aerosaccus SK60 | [553217](https://www.ncbi.nlm.nih.gov/Taxonomy/Browser/wwwtax.cgi?id=553217) | - |
| Staphylococcus lugdunensis | [28035](https://www.ncbi.nlm.nih.gov/Taxonomy/Browser/wwwtax.cgi?id=28035) | + |
| Paenibacillus sophorae S27 | [682957](https://www.ncbi.nlm.nih.gov/Taxonomy/Browser/wwwtax.cgi?id=682957) | + |
| Staphylococcus aureus subsp. aureus CO-98 | [904802](https://www.ncbi.nlm.nih.gov/Taxonomy/Browser/wwwtax.cgi?id=904802) | + |
| Acinetobacter baumannii | [470](https://www.ncbi.nlm.nih.gov/Taxonomy/Browser/wwwtax.cgi?id=470) | - |
| Rothia | [32207](https://www.ncbi.nlm.nih.gov/Taxonomy/Browser/wwwtax.cgi?id=32207) | + |
| Staphylococcus | [1279](https://www.ncbi.nlm.nih.gov/Taxonomy/Browser/wwwtax.cgi?id=1279) | + |
| Dermacoccus | [57495](https://www.ncbi.nlm.nih.gov/Taxonomy/Browser/wwwtax.cgi?id=57495) | + |
| Pantoea sp. 3.5.1 | [1522060](https://www.ncbi.nlm.nih.gov/Taxonomy/Browser/wwwtax.cgi?id=1522060) | - |
| Listeria monocytogenes LM201 | [1417986](https://www.ncbi.nlm.nih.gov/Taxonomy/Browser/wwwtax.cgi?id=1417986) | + |
| Kocuria atrinae C3-8 | [1179225](https://www.ncbi.nlm.nih.gov/Taxonomy/Browser/wwwtax.cgi?id=1179225) | + |
| Corynebacterium ihumii | [1232427](https://www.ncbi.nlm.nih.gov/Taxonomy/Browser/wwwtax.cgi?id=1232427) | + |
| Ralstonia sp. AU12-08 | [1235457](https://www.ncbi.nlm.nih.gov/Taxonomy/Browser/wwwtax.cgi?id=1235457) | - |
| Staphylococcus capitis C87 | [435838](https://www.ncbi.nlm.nih.gov/Taxonomy/Browser/wwwtax.cgi?id=435838) | + |
| Clostridiales | [186802](https://www.ncbi.nlm.nih.gov/Taxonomy/Browser/wwwtax.cgi?id=186802) | + |
| Kocuria marina | [223184](https://www.ncbi.nlm.nih.gov/Taxonomy/Browser/wwwtax.cgi?id=223184) | + |
| Curtobacterium sp. S6 | [1479623](https://www.ncbi.nlm.nih.gov/Taxonomy/Browser/wwwtax.cgi?id=1479623) | + |
| Acinetobacter calcoaceticus subsp. anitratus XM1570 | [1235833](https://www.ncbi.nlm.nih.gov/Taxonomy/Browser/wwwtax.cgi?id=1235833) | - |
| Staphylococcus caprae M23864:W1 | [525378](https://www.ncbi.nlm.nih.gov/Taxonomy/Browser/wwwtax.cgi?id=525378) | + |
| Staphylococcus xylosus | [1288](https://www.ncbi.nlm.nih.gov/Taxonomy/Browser/wwwtax.cgi?id=1288) | + |
| Staphylococcus schweitzeri | [1654388](https://www.ncbi.nlm.nih.gov/Taxonomy/Browser/wwwtax.cgi?id=1654388) | + |
| Staphylococcus argenteus | [985002](https://www.ncbi.nlm.nih.gov/Taxonomy/Browser/wwwtax.cgi?id=985002) | + |
| Staphylococcus simiae CCM 7213 | [911238](https://www.ncbi.nlm.nih.gov/Taxonomy/Browser/wwwtax.cgi?id=911238) | + |
| Brochothrix | [2755](https://www.ncbi.nlm.nih.gov/Taxonomy/Browser/wwwtax.cgi?id=2755) | + |
| Streptococcus agalactiae 18RS21 | [342613](https://www.ncbi.nlm.nih.gov/Taxonomy/Browser/wwwtax.cgi?id=342613) | + |
| Streptococcus | [1301](https://www.ncbi.nlm.nih.gov/Taxonomy/Browser/wwwtax.cgi?id=1301) | + |
| Lactobacillus | [1578](https://www.ncbi.nlm.nih.gov/Taxonomy/Browser/wwwtax.cgi?id=1578) | + |
| Granulicatella | [117563](https://www.ncbi.nlm.nih.gov/Taxonomy/Browser/wwwtax.cgi?id=117563) | + |
| Christensenella | [990721](https://www.ncbi.nlm.nih.gov/Taxonomy/Browser/wwwtax.cgi?id=990721) | - |
| Thermoanaerobacterium | [28895](https://www.ncbi.nlm.nih.gov/Taxonomy/Browser/wwwtax.cgi?id=28895) | + |
| Parvimonas | [543311](https://www.ncbi.nlm.nih.gov/Taxonomy/Browser/wwwtax.cgi?id=543311) | + |
| Kocuria | [57493](https://www.ncbi.nlm.nih.gov/Taxonomy/Browser/wwwtax.cgi?id=57493) | + |
| Micrococcaceae | [1268](https://www.ncbi.nlm.nih.gov/Taxonomy/Browser/wwwtax.cgi?id=1268) | + |
| Propionibacterium acnes HL087PA2 | [765082](https://www.ncbi.nlm.nih.gov/Taxonomy/Browser/wwwtax.cgi?id=765082) | + |
| Propionibacterium sp. 5_U_42AFAA | [450748](https://www.ncbi.nlm.nih.gov/Taxonomy/Browser/wwwtax.cgi?id=450748) | + |
| Corynebacterium jeikeium | [38289](https://www.ncbi.nlm.nih.gov/Taxonomy/Browser/wwwtax.cgi?id=38289) | + |
| Corynebacterium ureicelerivorans | [401472](https://www.ncbi.nlm.nih.gov/Taxonomy/Browser/wwwtax.cgi?id=401472) | + |
| Segniliparus | [286801](https://www.ncbi.nlm.nih.gov/Taxonomy/Browser/wwwtax.cgi?id=286801) | + |
| Glycomyces | [58113](https://www.ncbi.nlm.nih.gov/Taxonomy/Browser/wwwtax.cgi?id=58113) | + |
| Acinetobacter calcoaceticus/baumannii complex | [909768](https://www.ncbi.nlm.nih.gov/Taxonomy/Browser/wwwtax.cgi?id=909768) | - |
| Acinetobacter nosocomialis Ab22222 | [1183155](https://www.ncbi.nlm.nih.gov/Taxonomy/Browser/wwwtax.cgi?id=1183155) | - |
| Acinetobacter ursingii DSM 16037 = CIP 107286 | [981336](https://www.ncbi.nlm.nih.gov/Taxonomy/Browser/wwwtax.cgi?id=981336) | - |
| Acinetobacter oleivorans | [1148157](https://www.ncbi.nlm.nih.gov/Taxonomy/Browser/wwwtax.cgi?id=1148157) | - |
| Pseudomonas aeruginosa | [287](https://www.ncbi.nlm.nih.gov/Taxonomy/Browser/wwwtax.cgi?id=287) | - |
| Escherichia coli MS 116-1 | [749538](https://www.ncbi.nlm.nih.gov/Taxonomy/Browser/wwwtax.cgi?id=749538) | - |
| Salmonella enterica subsp. enterica | [59201](https://www.ncbi.nlm.nih.gov/Taxonomy/Browser/wwwtax.cgi?id=59201) | - |
| Thiomicrorhabdus | [2039723](https://www.ncbi.nlm.nih.gov/Taxonomy/Browser/wwwtax.cgi?id=2039723) | - |
| Nevskia | [64001](https://www.ncbi.nlm.nih.gov/Taxonomy/Browser/wwwtax.cgi?id=64001) | - |
| Afipia | [1033](https://www.ncbi.nlm.nih.gov/Taxonomy/Browser/wwwtax.cgi?id=1033) | - |
| Bordetella pertussis B1920 | [743278](https://www.ncbi.nlm.nih.gov/Taxonomy/Browser/wwwtax.cgi?id=743278) | - |
| Campylobacter | [194](https://www.ncbi.nlm.nih.gov/Taxonomy/Browser/wwwtax.cgi?id=194) | - |
| Capnocytophaga | [1016](https://www.ncbi.nlm.nih.gov/Taxonomy/Browser/wwwtax.cgi?id=1016) | - |
| Porphyromonas | [836](https://www.ncbi.nlm.nih.gov/Taxonomy/Browser/wwwtax.cgi?id=836) | - |

**Sample 2 – Bacteria Raw Data (Strain Level) - Total**

| **Name** | **Tax ID** | **Gram-Stain** |
| --- | --- | --- |
| Staphylococcus aureus | [1280](https://www.ncbi.nlm.nih.gov/Taxonomy/Browser/wwwtax.cgi?id=1280) | + |
| Staphylococcus warneri VCU121 | [904338](https://www.ncbi.nlm.nih.gov/Taxonomy/Browser/wwwtax.cgi?id=904338) | + |
| Staphylococcus pasteuri SP1 | [1276282](https://www.ncbi.nlm.nih.gov/Taxonomy/Browser/wwwtax.cgi?id=1276282) | + |
| Staphylococcus capitis QN1 | [1189311](https://www.ncbi.nlm.nih.gov/Taxonomy/Browser/wwwtax.cgi?id=1189311) | + |
| Staphylococcus haemolyticus R1P1 | [1134914](https://www.ncbi.nlm.nih.gov/Taxonomy/Browser/wwwtax.cgi?id=1134914) | + |
| Acinetobacter baumannii | [470](https://www.ncbi.nlm.nih.gov/Taxonomy/Browser/wwwtax.cgi?id=470) | - |
| Staphylococcus epidermidis NIH051668 | [1155131](https://www.ncbi.nlm.nih.gov/Taxonomy/Browser/wwwtax.cgi?id=1155131) | + |
| Pseudomonas psychrotolerans L19 | [1112217](https://www.ncbi.nlm.nih.gov/Taxonomy/Browser/wwwtax.cgi?id=1112217) | - |
| Staphylococcus sp. TE8 | [1472720](https://www.ncbi.nlm.nih.gov/Taxonomy/Browser/wwwtax.cgi?id=1472720) | + |
| Staphylococcus hominis SK119 | [629742](https://www.ncbi.nlm.nih.gov/Taxonomy/Browser/wwwtax.cgi?id=629742) | + |
| Micrococcus luteus | [1270](https://www.ncbi.nlm.nih.gov/Taxonomy/Browser/wwwtax.cgi?id=1270) | + |
| Pseudomonas | [286](https://www.ncbi.nlm.nih.gov/Taxonomy/Browser/wwwtax.cgi?id=286) | - |
| Pseudomonas oryzihabitans NBRC 102199 | [1215113](https://www.ncbi.nlm.nih.gov/Taxonomy/Browser/wwwtax.cgi?id=1215113) | - |
| Staphylococcus lugdunensis | [28035](https://www.ncbi.nlm.nih.gov/Taxonomy/Browser/wwwtax.cgi?id=28035) | + |
| Pseudomonas sp. MOIL14HWK12:I2 | [1033994](https://www.ncbi.nlm.nih.gov/Taxonomy/Browser/wwwtax.cgi?id=1033994) | - |
| Staphylococcus cohnii hu-01 | [1415164](https://www.ncbi.nlm.nih.gov/Taxonomy/Browser/wwwtax.cgi?id=1415164) | + |
| Staphylococcus | [1279](https://www.ncbi.nlm.nih.gov/Taxonomy/Browser/wwwtax.cgi?id=1279) | + |
| Kocuria palustris PEL | [1236550](https://www.ncbi.nlm.nih.gov/Taxonomy/Browser/wwwtax.cgi?id=1236550) | + |
| Staphylococcus saprophyticus | [29385](https://www.ncbi.nlm.nih.gov/Taxonomy/Browser/wwwtax.cgi?id=29385) | + |
| Staphylococcus schweitzeri | [1654388](https://www.ncbi.nlm.nih.gov/Taxonomy/Browser/wwwtax.cgi?id=1654388) | + |
| Staphylococcus argenteus | [985002](https://www.ncbi.nlm.nih.gov/Taxonomy/Browser/wwwtax.cgi?id=985002) | + |
| Pseudomonas aeruginosa | [287](https://www.ncbi.nlm.nih.gov/Taxonomy/Browser/wwwtax.cgi?id=287) | - |
| Ralstonia sp. AU12-08 | [1235457](https://www.ncbi.nlm.nih.gov/Taxonomy/Browser/wwwtax.cgi?id=1235457) | - |
| Staphylococcus caprae M23864:W1 | [525378](https://www.ncbi.nlm.nih.gov/Taxonomy/Browser/wwwtax.cgi?id=525378) | + |
| Staphylococcus xylosus NJ | [1262650](https://www.ncbi.nlm.nih.gov/Taxonomy/Browser/wwwtax.cgi?id=1262650) | + |
| Staphylococcus simiae CCM 7213 | [911238](https://www.ncbi.nlm.nih.gov/Taxonomy/Browser/wwwtax.cgi?id=911238) | + |
| Exiguobacterium | [33986](https://www.ncbi.nlm.nih.gov/Taxonomy/Browser/wwwtax.cgi?id=33986) | + |
| Kurthia | [1649](https://www.ncbi.nlm.nih.gov/Taxonomy/Browser/wwwtax.cgi?id=1649) | + |
| Brochothrix | [2755](https://www.ncbi.nlm.nih.gov/Taxonomy/Browser/wwwtax.cgi?id=2755) | + |
| Lactobacillus | [1578](https://www.ncbi.nlm.nih.gov/Taxonomy/Browser/wwwtax.cgi?id=1578) | + |
| Granulicatella | [117563](https://www.ncbi.nlm.nih.gov/Taxonomy/Browser/wwwtax.cgi?id=117563) | + |
| Streptococcus agalactiae 18RS21 | [342613](https://www.ncbi.nlm.nih.gov/Taxonomy/Browser/wwwtax.cgi?id=342613) | + |
| Clostridiales | [186802](https://www.ncbi.nlm.nih.gov/Taxonomy/Browser/wwwtax.cgi?id=186802) | + |
| Christensenella | [990721](https://www.ncbi.nlm.nih.gov/Taxonomy/Browser/wwwtax.cgi?id=990721) | - |
| Sulfobacillus | [28033](https://www.ncbi.nlm.nih.gov/Taxonomy/Browser/wwwtax.cgi?id=28033) | + |
| Thermoanaerobacterium | [28895](https://www.ncbi.nlm.nih.gov/Taxonomy/Browser/wwwtax.cgi?id=28895) | + |
| Parvimonas | [543311](https://www.ncbi.nlm.nih.gov/Taxonomy/Browser/wwwtax.cgi?id=543311) | + |
| Rothia | [32207](https://www.ncbi.nlm.nih.gov/Taxonomy/Browser/wwwtax.cgi?id=32207) | + |
| Propionibacteriaceae | [31957](https://www.ncbi.nlm.nih.gov/Taxonomy/Browser/wwwtax.cgi?id=31957) | + |
| Propionibacterium sp. KPL1844 | [1203573](https://www.ncbi.nlm.nih.gov/Taxonomy/Browser/wwwtax.cgi?id=1203573) | + |
| Pseudonocardia sp. P2 | [882856](https://www.ncbi.nlm.nih.gov/Taxonomy/Browser/wwwtax.cgi?id=882856) | + |
| Salmonella enterica subsp. enterica serovar Typhimurium | [90371](https://www.ncbi.nlm.nih.gov/Taxonomy/Browser/wwwtax.cgi?id=90371) | - |
| Bradyrhizobiaceae | [41294](https://www.ncbi.nlm.nih.gov/Taxonomy/Browser/wwwtax.cgi?id=41294) | - |
| Sphingomonas | [13687](https://www.ncbi.nlm.nih.gov/Taxonomy/Browser/wwwtax.cgi?id=13687) | - |
| Anaeromyxobacter | [161492](https://www.ncbi.nlm.nih.gov/Taxonomy/Browser/wwwtax.cgi?id=161492) | - |
| Dictyoglomus | [13](https://www.ncbi.nlm.nih.gov/Taxonomy/Browser/wwwtax.cgi?id=13) | - |
| Capnocytophaga | [1016](https://www.ncbi.nlm.nih.gov/Taxonomy/Browser/wwwtax.cgi?id=1016) | - |
| Porphyromonas | [836](https://www.ncbi.nlm.nih.gov/Taxonomy/Browser/wwwtax.cgi?id=836) | - |
| Candidatus Methanoperedens nitroreducens | [1392998](https://www.ncbi.nlm.nih.gov/Taxonomy/Browser/wwwtax.cgi?id=1392998) | - |

**Sample 3 – Bacteria Raw Data (Strain Level) - Total**

| **Name** | **Tax ID** | **Gram-Stain** |
| --- | --- | --- |
| Staphylococcus epidermidis | [1282](https://www.ncbi.nlm.nih.gov/Taxonomy/Browser/wwwtax.cgi?id=1282) | + |
| Staphylococcus aureus DAR3599 | [1422144](https://www.ncbi.nlm.nih.gov/Taxonomy/Browser/wwwtax.cgi?id=1422144) | + |
| Micrococcus luteus SK58 | [596312](https://www.ncbi.nlm.nih.gov/Taxonomy/Browser/wwwtax.cgi?id=596312) | + |
| Staphylococcus sp. MDS7B | [1209359](https://www.ncbi.nlm.nih.gov/Taxonomy/Browser/wwwtax.cgi?id=1209359) | + |
| Staphylococcus capitis | [29388](https://www.ncbi.nlm.nih.gov/Taxonomy/Browser/wwwtax.cgi?id=29388) | + |
| Staphylococcus haemolyticus DNF00585 | [1401071](https://www.ncbi.nlm.nih.gov/Taxonomy/Browser/wwwtax.cgi?id=1401071) | + |
| Staphylococcus warneri L37603 | [596319](https://www.ncbi.nlm.nih.gov/Taxonomy/Browser/wwwtax.cgi?id=596319) | + |
| Staphylococcus hominis subsp. hominis ZBW5 | [1185421](https://www.ncbi.nlm.nih.gov/Taxonomy/Browser/wwwtax.cgi?id=1185421) | + |
| Enhydrobacter aerosaccus SK60 | [553217](https://www.ncbi.nlm.nih.gov/Taxonomy/Browser/wwwtax.cgi?id=553217) | - |
| Staphylococcus pasteuri SP1 | [1276282](https://www.ncbi.nlm.nih.gov/Taxonomy/Browser/wwwtax.cgi?id=1276282) | + |
| Paenibacillus sophorae S27 | [682957](https://www.ncbi.nlm.nih.gov/Taxonomy/Browser/wwwtax.cgi?id=682957) | + |
| Kocuria palustris PEL | [1236550](https://www.ncbi.nlm.nih.gov/Taxonomy/Browser/wwwtax.cgi?id=1236550) | + |
| Ralstonia sp. AU12-08 | [1235457](https://www.ncbi.nlm.nih.gov/Taxonomy/Browser/wwwtax.cgi?id=1235457) | - |
| Staphylococcus schweitzeri | [1654388](https://www.ncbi.nlm.nih.gov/Taxonomy/Browser/wwwtax.cgi?id=1654388) | + |
| Staphylococcus argenteus | [985002](https://www.ncbi.nlm.nih.gov/Taxonomy/Browser/wwwtax.cgi?id=985002) | + |
| Acinetobacter baumannii EGD-HP18 | [1358412](https://www.ncbi.nlm.nih.gov/Taxonomy/Browser/wwwtax.cgi?id=1358412) | - |
| Propionibacterium acnes HL025PA1 | [765089](https://www.ncbi.nlm.nih.gov/Taxonomy/Browser/wwwtax.cgi?id=765089) | + |
| Staphylococcus massiliensis | [555791](https://www.ncbi.nlm.nih.gov/Taxonomy/Browser/wwwtax.cgi?id=555791) | + |
| Listeria monocytogenes LM201 | [1417986](https://www.ncbi.nlm.nih.gov/Taxonomy/Browser/wwwtax.cgi?id=1417986) | + |
| Lactobacillus delbrueckii subsp. bulgaricus | [1585](https://www.ncbi.nlm.nih.gov/Taxonomy/Browser/wwwtax.cgi?id=1585) | + |
| Staphylococcus saprophyticus subsp. saprophyticus KACC 16562 | [1131257](https://www.ncbi.nlm.nih.gov/Taxonomy/Browser/wwwtax.cgi?id=1131257) | + |
| Staphylococcus | [1279](https://www.ncbi.nlm.nih.gov/Taxonomy/Browser/wwwtax.cgi?id=1279) | + |
| Staphylococcus lugdunensis M23590 | [525377](https://www.ncbi.nlm.nih.gov/Taxonomy/Browser/wwwtax.cgi?id=525377) | + |
| Propionibacterium sp. MB3007 | [1410615](https://www.ncbi.nlm.nih.gov/Taxonomy/Browser/wwwtax.cgi?id=1410615) | + |
| Isosphaeraceae | [1763524](https://www.ncbi.nlm.nih.gov/Taxonomy/Browser/wwwtax.cgi?id=1763524) | - |
| Staphylococcus simiae CCM 7213 | [911238](https://www.ncbi.nlm.nih.gov/Taxonomy/Browser/wwwtax.cgi?id=911238) | + |
| Staphylococcus caprae M23864:W1 | [525378](https://www.ncbi.nlm.nih.gov/Taxonomy/Browser/wwwtax.cgi?id=525378) | + |
| Staphylococcus xylosus | [1288](https://www.ncbi.nlm.nih.gov/Taxonomy/Browser/wwwtax.cgi?id=1288) | + |
| Streptococcus agalactiae 18RS21 | [342613](https://www.ncbi.nlm.nih.gov/Taxonomy/Browser/wwwtax.cgi?id=342613) | + |
| Clostridiales | [186802](https://www.ncbi.nlm.nih.gov/Taxonomy/Browser/wwwtax.cgi?id=186802) | + |
| Bacillaceae | [186817](https://www.ncbi.nlm.nih.gov/Taxonomy/Browser/wwwtax.cgi?id=186817) | + |
| Streptococcus thermophilus LMG 18311 | [264199](https://www.ncbi.nlm.nih.gov/Taxonomy/Browser/wwwtax.cgi?id=264199) | + |
| Lactobacillus | [1578](https://www.ncbi.nlm.nih.gov/Taxonomy/Browser/wwwtax.cgi?id=1578) | + |
| Granulicatella | [117563](https://www.ncbi.nlm.nih.gov/Taxonomy/Browser/wwwtax.cgi?id=117563) | + |
| Thermoanaerobacterium | [28895](https://www.ncbi.nlm.nih.gov/Taxonomy/Browser/wwwtax.cgi?id=28895) | + |
| Kytococcus sedentarius DSM 20547 | [478801](https://www.ncbi.nlm.nih.gov/Taxonomy/Browser/wwwtax.cgi?id=478801) | + |
| Cutibacterium granulosum TM11 | [1292373](https://www.ncbi.nlm.nih.gov/Taxonomy/Browser/wwwtax.cgi?id=1292373) | + |
| Blastococcus | [38501](https://www.ncbi.nlm.nih.gov/Taxonomy/Browser/wwwtax.cgi?id=38501) | + |
| Ralstonia | [48736](https://www.ncbi.nlm.nih.gov/Taxonomy/Browser/wwwtax.cgi?id=48736) | - |
| Afipia birgiae 34632 | [1197906](https://www.ncbi.nlm.nih.gov/Taxonomy/Browser/wwwtax.cgi?id=1197906) | - |
| Bradyrhizobiaceae | [41294](https://www.ncbi.nlm.nih.gov/Taxonomy/Browser/wwwtax.cgi?id=41294) | - |
| Capnocytophaga | [1016](https://www.ncbi.nlm.nih.gov/Taxonomy/Browser/wwwtax.cgi?id=1016) | - |
| Staphylococcus gallinarum | [1293](https://www.ncbi.nlm.nih.gov/Taxonomy/Browser/wwwtax.cgi?id=1293) | + |
| Bacillus cereus F65185 | [526989](https://www.ncbi.nlm.nih.gov/Taxonomy/Browser/wwwtax.cgi?id=526989) | + |
| Exiguobacterium | [33986](https://www.ncbi.nlm.nih.gov/Taxonomy/Browser/wwwtax.cgi?id=33986) | + |
| Brochothrix | [2755](https://www.ncbi.nlm.nih.gov/Taxonomy/Browser/wwwtax.cgi?id=2755) | + |
| Carnobacterium | [2747](https://www.ncbi.nlm.nih.gov/Taxonomy/Browser/wwwtax.cgi?id=2747) | + |
| Enterococcus | [1350](https://www.ncbi.nlm.nih.gov/Taxonomy/Browser/wwwtax.cgi?id=1350) | + |
| Christensenella | [990721](https://www.ncbi.nlm.nih.gov/Taxonomy/Browser/wwwtax.cgi?id=990721) | - |
| Parvimonas | [543311](https://www.ncbi.nlm.nih.gov/Taxonomy/Browser/wwwtax.cgi?id=543311) | + |
| Kocuria | [57493](https://www.ncbi.nlm.nih.gov/Taxonomy/Browser/wwwtax.cgi?id=57493) | + |
| Micrococcaceae | [1268](https://www.ncbi.nlm.nih.gov/Taxonomy/Browser/wwwtax.cgi?id=1268) | + |
| Corynebacterium kroppenstedtii DSM 44385 | [645127](https://www.ncbi.nlm.nih.gov/Taxonomy/Browser/wwwtax.cgi?id=645127) | + |
| Glycomyces | [58113](https://www.ncbi.nlm.nih.gov/Taxonomy/Browser/wwwtax.cgi?id=58113) | + |
| Solirubrobacter | [207599](https://www.ncbi.nlm.nih.gov/Taxonomy/Browser/wwwtax.cgi?id=207599) | + |
| Pseudomonas | [286](https://www.ncbi.nlm.nih.gov/Taxonomy/Browser/wwwtax.cgi?id=286) | - |
| Afipia | [1033](https://www.ncbi.nlm.nih.gov/Taxonomy/Browser/wwwtax.cgi?id=1033) | - |
| Mesorhizobium | [68287](https://www.ncbi.nlm.nih.gov/Taxonomy/Browser/wwwtax.cgi?id=68287) | - |
| Methylobacterium | [407](https://www.ncbi.nlm.nih.gov/Taxonomy/Browser/wwwtax.cgi?id=407) | - |
| Dictyoglomus | [13](https://www.ncbi.nlm.nih.gov/Taxonomy/Browser/wwwtax.cgi?id=13) | - |
| Porphyromonas | [836](https://www.ncbi.nlm.nih.gov/Taxonomy/Browser/wwwtax.cgi?id=836) | - |
